# Antibacterial Drug Discovery: Deep Learning Successes and Challenges through the Structural Biology Lens

**DOI:** 10.34133/csbj.0008

**Published:** 2026-03-24

**Authors:** Jameel M. Abduljalil, Sandro F. Ataide, Ann H. Kwan

**Affiliations:** School of Life and Environmental Sciences, The University of Sydney, Sydney, NSW, Australia.

## Abstract

The continuous rise of multidrug-resistant pathogens necessitates an urgent need for new antibiotics, yet innovation in antibiotic discovery has largely stalled since the 1980s. In traditional drug development, around 90% of candidate molecules fail at the preclinical stage or phase I of trials due to toxicity or lack of efficacy. Effective antibiotic discovery must overcome a set of microbiological challenges: selective bacterial targeting, penetration of complex cell envelopes, and evasion of diverse resistance mechanisms. Recent advances in deep learning (DL) offer promising opportunities to address these challenges. DL can help identify and characterize new bacterial targets, predict accurate 3-dimensional structures, assess druggability, and discover lead molecules with antibiotic potential. Generative models further enable the de novo design of candidates with optimized pharmacokinetics and safety profiles, potentially resolving long-standing toxicity issues. These technologies streamline labor-intensive screening and boost efficiency in the drug discovery pipeline. However, DL methods need to be applied judiciously. Their effectiveness depends on appropriate model selection, high-quality training data, and careful interpretation of predictions particularly when predicting properties for novel microbial targets. This review provides a timely and critical analysis of DL applications in antibacterial hit discovery through the lens of structural biology, offering structural biologists a road map for integrating these tools into antibiotic discovery workflows to help combat antimicrobial resistance.

## Graphical Abstract

**Figure:**
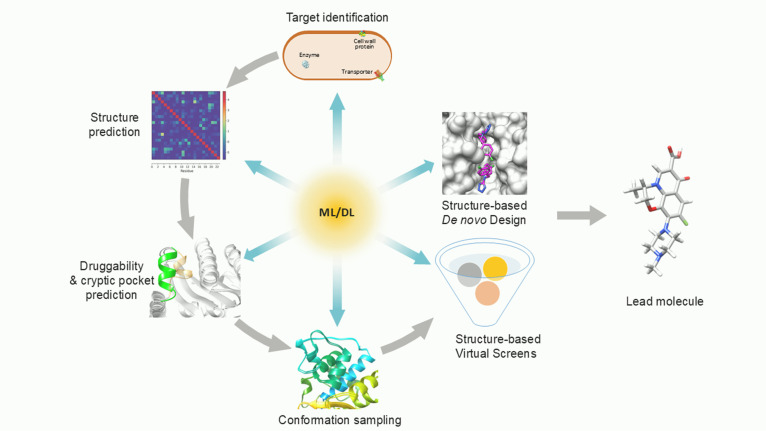

## Introduction

The majority of antibiotics currently used in clinical practice were developed between the 1940s and the 1970s [1]. These drugs target a limited set of bacterial structures and processes, including the cell wall, ribosomes, and the machinery for transcription and DNA replication. Beyond the propagation of naturally occurring resistance genes via mobile genetic elements [2], the use of antibiotics imposes selective pressures that have catalyzed the emergence and diversification of bacterial resistance mechanisms [3]. These include (a) drug-inactivating enzymes such as β-lactamases and aminoglycoside- or macrolide-modifying enzymes, (b) reduced membrane permeability through the regulation of porin expression, (c) active efflux of antibiotics via pumps like the multidrug resistance AcrAB–TolC and RND efflux systems, (d) target protection as seen in tetracycline resistance via the ribosome-protecting proteins tet(M) and tet(O), and (e) alterations in drug targets through mutations or chemical modifications that reduce binding affinity [4].

The global estimates of mortalities associated with drug-resistant bacterial pathogens are alarming [5]. In the past 3 decades (1990 to 2021), deaths among the elderly due to antibiotic-resistant infections increased by approximately 80%, with an estimated 5 million deaths associated with antimicrobial resistance in 2021, including 1.14 million directly attributable to antibiotic-resistant pathogens. In 2024, the American Centers for Disease Control and Prevention reported that antimicrobial-resistant infections caused by 6 high-priority pathogens (carbapenem-resistant Enterobacterales, carbapenem-resistant *Acinetobacter*, methicillin-resistant *Staphylococcus aureus*, vancomycin-resistant *Enterococcus*, extended-spectrum β-lactamase-producing Enterobacterales, and multidrug-resistant *Pseudomonas aeruginosa*) increased by ~20% during the COVID-19 pandemic, peaking in 2021 and remaining above baseline levels in 2022 [6]. Projections estimate that by 2050, annual deaths associated with antibiotic resistance could exceed 8 million, with nearly 2 million directly attributable to drug-resistant infections [5].

Despite the growing threat of multidrug-resistant pathogens, the clinical approval of truly novel antibiotics has remained limited over the last 5 decades [7]. For example, prior to the recent Food and Drug Administration (FDA) approval of gepotidacin, a first-in-class triazaacenaphthylene antibiotic, in March 2025, the last new class effective against gram-positive and gram-negative bacteria to reach clinical use was the fluoroquinolones in 1986. Although members of the new classes oxazolidinone and lipopeptide were approved in 2000 and 2003 by the FDA, they are ineffective against gram-negative bacteria. While numerous novel scaffolds are currently under investigation [8], development efforts have largely yielded structural modifications of existing antibiotics, such as revised β-lactamase inhibitors rather than truly novel antibiotics [9]. Most of the recently approved antibiotics are derivatives of existing drugs and, while chemically modified, still act on the same bacterial targets as their predecessors to which resistance has already evolved.

The integration of deep learning (DL) into antibiotic drug discovery presents transformative opportunities by enabling the analysis of vast and complex biological datasets to uncover novel antibiotic compounds, targets, and pharmacokinetics. Traditional antibiotic discovery methods rely heavily on labor-intensive screening and serendipitous findings, whereas DL models can predict antimicrobial activity, identify previously overlooked molecular structures, and optimize lead compounds with enhanced efficacy and reduced resistance potential [10].

The number of DL models developed and published for various tasks in structural biology and drug discovery is increasing rapidly, yet experimental validation is limited to a small percentage. Although many excellent reviews have addressed the capabilities and technicalities of DL in the discovery of new drugs [10–16], the examination of DL models from a structural biology perspective is seen only as short sections scattered in different articles. This review starts with an overview of the commonly encountered DL principles and architectures followed by an examination of the potential applications of DL in the identification of novel antibiotic targets, subsequently exploring its role in generating accurate 3-dimensional (3D) models of the targets in complex with other macromolecules, and different approaches to identify potential small-molecule or peptide hits. Emerging DL-based strategies for detecting cryptic pockets are discussed, emphasizing the early-stage nature of this research area. The new avenues for targeting bacterial structures, such as RNA-binding proteins (RBPs), and advancements in de novo design approaches for antibiotic discovery are critically reviewed. State-of-the-art pipelines for the co-folding of receptors and their potential modulators are also analyzed, with particular attention to their successes as well as limitations. In the context of hit discovery, we focused only on the DL models that led to experimentally validated small-molecule or antimicrobial peptides (AMPs).

## The Discovery of Antibiotics: Challenges and Promise

**Fig. 1.**
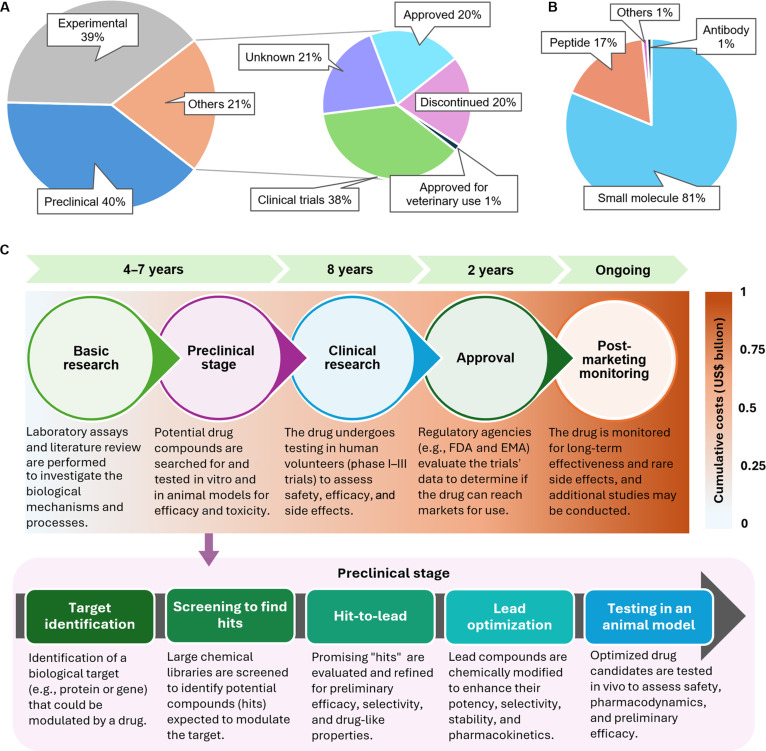
Status of all antibiotics and the drug discovery pipeline. The numbers in (A) and (B) are based on the entries of an antibiotic database [17] (*n* = 3,453) (last update 31 May 2025). A molecule is classified as “approved” if its corresponding database entry indicates approval by regulatory authorities such as the Food and Drug Administration (FDA), the European Union, Japan, Taiwan, or “approved by several countries”, even if its approval has been discontinued in certain regions. The “discontinued” category includes molecules whose labels indicate discontinued or withdrawn or are no longer approved in most or many countries. Molecules are designated as “unknown” if they lack any label or if they are approved only outside of the United States, the European Union, Japan, and Taiwan. The “clinical trials” category comprises molecules currently undergoing any phase of clinical trials. Molecules in the remaining categories retained their original labels in the database. The curated dataset is available as Supplementary File in Excel format. (C) The general processes in the pipeline of drug discovery. The lower panel details the substages of preclinical drug discovery where deep learning (DL) is transforming the approach. EMA, European Medicines Agency.

Since the discovery of penicillin nearly a century ago, antibiotics, mostly small molecules (molecular weight < 1,000 Da) (Fig. 1), have become indispensable to modern medicine. The period from the 1940s to the 1970s witnessed substantial antibiotic discoveries, with most current classes of antibiotics being identified from natural sources during that period [17]. Current statistics indicate that only 5% to 7% of all candidate molecules discovered from the 1920s until today have been approved for human use (Fig. 1A). It is noteworthy that this fraction may be overestimated, as the majority of approved antibiotics are chemically modified versions of parent drugs but intended to counteract bacterial resistance to the parent molecule.

Small molecules have the potential for oral bioavailability and the ability to cross the blood–brain barrier, particularly with the wealth of knowledge from medicinal chemistry. Such accumulated knowledge facilitates systematic optimization of physiochemical properties to improve pharmacokinetics and potency [18]. Small molecules, however, have low specificity and potency, which are major issues faced in traditional drug discovery campaigns. Peptides, on the other hand, gained more recent attention as antimicrobial molecules in drug discovery campaigns due to their high specificity. Currently, the design of peptides with high specificity have been a successful approach, especially for disrupting protein–protein interactions [19]. However, peptides face obstacles in terms of absorption, stability in cells, short half-life in blood, and potential immunogenicity [20].

Developing a new antibiotic from scratch to the market typically costs about US$1 billion and over a decade of research and development (Fig. 1C) [21]. The conventional approach of drug discovery when applied to antibiotic discovery is associated with a high rate of failure approaching 90% when most candidate molecules fail during phase I of clinical trials due to toxicity or lack of clinical efficacy [22]. One of the main challenges is the identification of novel targets that are essential to bacterial survival or pathogenicity but distinct from human homologs. Most existing antibiotics act on a single target or a narrow set of bacterial targets, many of which have already been extensively targeted, leaving few unexplored and viable avenues. In addition to the required safe pharmacokinetic profile, most antibiotics are intended to penetrate complex bacterial cell envelopes and remain active. Furthermore, candidate antibiotics must overcome diverse resistance mechanisms, such as efflux pumps or enzymatic inactivation, in addition to demonstrating selective toxicity against pathogens with minimal damage to commensal microbiota. Consequently, their discovery necessitates additional criteria that differ substantially from the criteria typically applied in the development of drugs for noninfectious diseases [23].

High-throughput screening (HTS) has been the cornerstone in drug discovery campaigns for the past 3 decades, and more than 90% of current clinical drug candidates originated from HTS [24]. Due to their resource-intensive nature, HTS screens are mostly conducted in the industry sector. Unfortunately, many pharmaceutical companies favor chronic disease treatments over antibiotic development due to longer treatment duration with a steady revenue [21]. Although HTS was well established in the early 1990s, no antibiotic has been discovered through this approach. One major limitation is the limited number of molecules (millions) that can, cost-effectively, be tested from the huge drug-like chemical space (estimated to be 10^60^ [25]). These constraints arise from several economic and practical challenges, including the need for large quantities of the purified protein target, efficient automation, prevention of aggregation, and overall experimental integrity [26]. These challenges associated with conventional methods are now believed to be addressable, to different extents, by DL methods that can significantly accelerate antibacterial lead discovery by analyzing vast datasets to identify novel targets, predict promising compounds, and optimize their properties [13].

## Artificial Intelligence in Structural Biology and Drug Discovery

Machine learning (ML) can be broadly defined as the capability of computers to identify and learn patterns without requiring explicit programming to do so. In its core, ML is a way for computers to learn patterns from data, similar to how you might fit a line to enzyme activity data to find a relationship. Instead of manually calculating the slope and the *y*-intercept, the computer automatically adjusts these values (a.k.a. parameters) to improve the fitting and consequently its predictions. While regression in enzyme experiments focuses on linear relationships, ML/DL can handle nonlinear, messy, or multidimensional data by iteratively refining the parameters (weights) of its models. DL is a specialized type of ML that uses multilayered “neural networks” inspired by neural architectures in the mammalian nervous system (brain and eyes). These networks can learn intricate patterns automatically, such as identifying shapes in microscope images, molecular property prediction, or predicting molecular structures.

Classical ML algorithms were primarily employed in quantitative structure–activity relationship modeling and other cheminformatics methods that focused on ligand-based strategies [27,28]. Their utility in discovering antibacterial lead compounds was reviewed elsewhere [29], yet they were constrained by limited data availability and insufficient computational power at that time. Indeed, many algorithms, such as decision trees, naive Bayes, multilayer perceptrons, and support vector machines, lacked graphics processing unit acceleration or struggled to scale efficiently with large, complex datasets.

### DL architectures adopted in structural biology

**Fig. 2.**
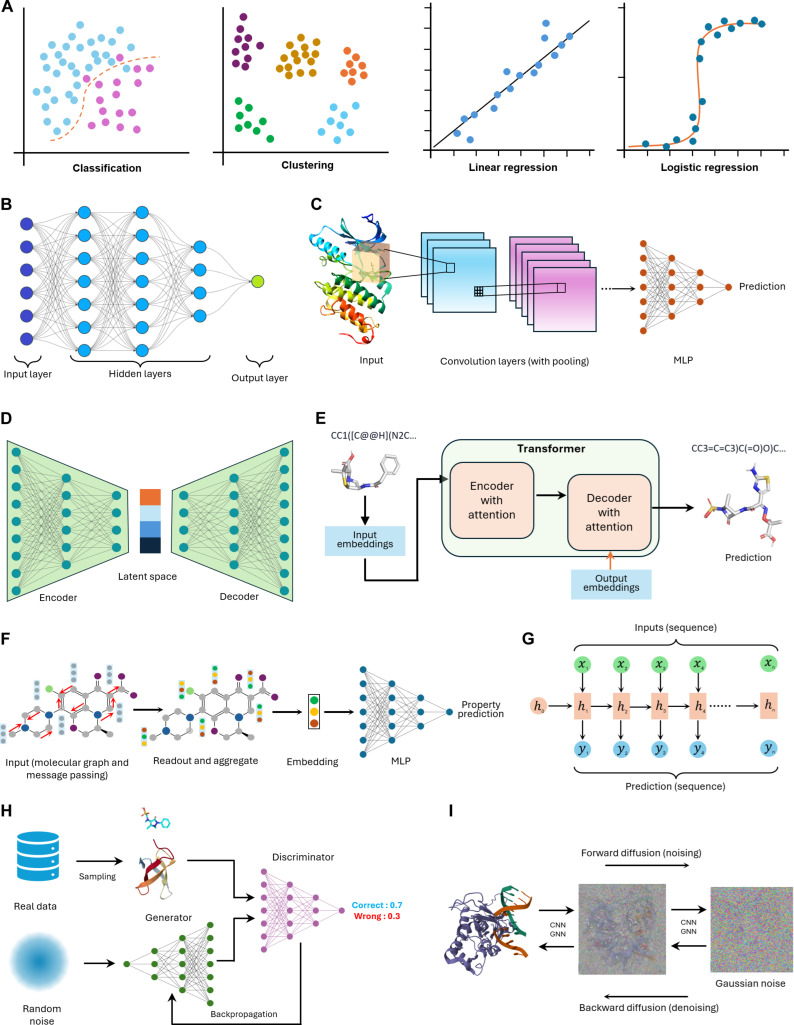
Common biological tasks and common neural network (NN) architectures. (A) Biological tasks that can usually be done via machine learning (ML) or DL models; however, complicated classifications and nonlinear regressions are modeled more efficiently via DL. (B) General architecture of a traditional NN (e.g., a multilayer perception [MLP]). (C) 3-dimensional (3D) convolutional NN as implemented in the Gnina software to predict the binding pose (conformation) and affinity of the docked small molecule. (D) Autoencoder architecture with a vector latent space. The vibrational autoencoder has similar architecture, but the latent space is a distribution that can be sampled for generative tasks. (E) A typical architecture of a transformer model. Decoders take previous output embeddings as input to condition each prediction on prior context during autoregressive generation. (F) A message-passing NN (a common type of graph NN) used to predict molecular properties in a classification or regression task. (G) Recurrent NN architecture. This type of model is useful in generating sequence data (e.g., peptides). The recurrent connections allow hidden states to retain and propagate information across time steps, enabling sequence modeling. (H) Generative adversarial networks. The discriminator is trained initially on real data, and then it is used as a detective to spot real data from the unreal data generated by a generator. The discriminator updates the generator via backpropagation, and the generator improves its predictions to mimic the real data as much as possible. This adversary nature has promising potential in de novo molecular design. (I). Diffusion model architecture. This model is trained by gradually adding and removing random noise (usually Gaussian) from the data of interest. Once the model manages to capture the distribution of the data, it can generate new data from a complete noise. CNN, convolutional neural network; GNN, graph neural network.

A plethora of DL models were developed to deal with specific data modalities and tasks that can be categorized as regression, classification, or clustering (Fig. 2A); however, certain architectures have established their usefulness in structural biology more than others. Based on their architecture, DL models can broadly be grouped into 3 main categories: discriminative, generative, and hybrid (Table 1). Discriminative DL models—such as feedforward neural networks (Fig. 2B)—learn decision boundaries to predict labels from data, focusing on the probability of labels or the value of an outcome *Y* given input *X*. These models, reviewed by Greener et al. [30] and Lecun et al. [31], excel in classification and regression tasks as in the classical ML models. Unlike classical ML models that often rely on hand-engineered features of structured data, discriminative DL models focus on learning the decision boundaries between different classes rather than modeling the underlying data distribution. One of the earliest successful adaptations of DL in structural biology was established around a decade ago [32] to score ligand pose interactions with a protein receptor, which was implemented later in the Gnina docking software [33].

**Table 1.** Major DL architectures in structural biology and drug discovery research

Category	Architecture	Concept	Pros	Cons	Applications	Examples
Discriminative	Convolutional neural network (CNN)	Learns spatial hierarchies by applying convolutional filters to local regions, enabling them to capture structural patterns in molecular data	Strong spatial feature extraction. Works well with grid-like data (e.g., protein structures and protein–ligand complexes)	Limited to local patterns unless combined with attention. Pooling can lead to loss of spatial information. Struggles with variable-length sequences	Protein structure prediction, ligand-binding affinity prediction, cryo-EM image analysis	AtomNet [148] Gnina (https://github.com/gnina/gnina) [129]
	Multilayer perceptron (MLP)	Fully connected FNNs that learn linear or nonlinear relationships between input features and target outputs	Versatile and can model complex nonlinear relationships between inputs and outputs. Serves as foundational models for various DL architectures	Lack inherent mechanisms to capture spatial or sequential dependencies. Requires large amounts of data for effective training	QSAR modeling and prediction of molecular properties. Used in combination with other models for feature extraction, regression, and classification tasks	Part of many pipelines such as Chemprop (https://github.com/chemprop/chemprop) [63] and DiffDock (https://github.com/gcorso/DiffDock) [54]
	Graph neural network (GNN)	Iteratively updates node representations by aggregating information from graph-structured neighbors	Handles molecular graphs, capturing relationships between atoms and bonds. Captures both local and global structural relationships	Computationally expensive for large graphs and complex molecular graphs. May struggle with long-range dependencies	Molecular property prediction, protein–protein interaction, and drug–target interaction. Applied in de novo drug design and virtual screening	PocketMiner (https://github.com/Mickdub/gvp/tree/pocket_pred) [100]
Hybrid	Variational autoencoder (VAE)	Encodes data into a latent probabilistic space and decodes them back, allowing for generative modeling of molecular structures with controlled variation	Capable of learning continuous latent representations, facilitating the generation of novel molecular structures. Useful in capturing the underlying distribution of complex biological data	Generated molecules may lack chemical validity without additional constraints	Molecular generation, latent space exploration, de novo drug design, molecular optimization, and protein engineering	TamGen (https://github.com/microsoft/TamGen) [52]
	Recurrent neural network (RNN)	Designed to handle sequential data, making them suitable for modeling biological sequences. Capable of capturing temporal dependencies in data	Handles sequential data well. Useful for autoregressive generation	Vanishing or exploding gradient problems, limiting their ability to capture long-range dependencies. Training can be time-consuming and computationally expensive	Modeling and generating SMILES strings for molecular structures. Applied in protein sequence analysis and prediction tasks	AMPd-Up (https://github.com/bcgsc/AMPd-Up) [135]
	Transformers (BERTs and GPTs)	Use self-attention mechanisms to model dependencies across sequences without recurrence, allowing deep contextual understanding of the input sequence	They capture global dependencies in sequences. Highly parallelizable. Pre-training enables transfer learning	High computational cost. Require large datasets for training	Protein structure prediction, molecular property prediction, and de novo drug design	AlphaFold2 (https://github.com/google-deepmind/alphafold) [36]
Generative	Generative adversarial networks (GANs)	Consist of a generator and a discriminator in competition, driving the generation of realistic synthetic new data	Generating high-quality, realistic molecular structures. Useful in augmenting datasets and exploring chemical space	Training can be unstable due to the adversarial nature of the model. Mode collapse can occur, leading to limited diversity in generated molecules	De novo drug design, molecular optimization, and virtual screening. Applied in generating novel compounds with desired properties	SeqGAN (https://github.com/LantaoYu/SeqGAN) [149] implemented in [138]
	Diffusion models	Learn to generate data by reversing a stochastic noise process, enabling the sampling of complex distributions	Prototype for learning data distributions through a diffusion process. Capable of generating high-fidelity and diverse molecular structures	Computationally intensive, requiring multiple iterations for generation. Relatively new in the field, with ongoing research to optimize performance	De novo molecular generation, structure prediction, drug design, and molecular docking	AlphaFold 3 (https://github.com/google-deepmind/alphafold3) [40] DiffDock (https://github.com/gcorso/DiffDock) [39]
	Autoregressive models	Predict each element in a sequence based on previous ones, capturing structured dependencies	Predict sequences one by one. Useful in capturing sequential dependencies. Useful in generating structured outputs	Generation is sequential, leading to slower inference times. Error accumulation can occur during generation, affecting output quality	Generation of SMILES strings for molecules, protein sequences, and other biological data	DiffSBDD (https://github.com/arneschneuing/DiffSBDD) [48]

Cryo-EM, cryogenic electron microscopy; FNNs, feedforward neural networks; QSAR, quantitative structure–activity relationship; SMILES, Simplified Molecular Input Line Entry System; BERTs, Bidirectional Encoder Representations from Transformers; GPTs, generative pre-trained transformers

Two of the masterpieces in DL and its application in structural biology and modeling are the autoencoders and variational autoencoders (VAEs) (Fig. 2D), hybrid neural networks that are used for unsupervised learning and dimensionality reduction, but they differ significantly in their approach and objectives. A standard autoencoder learns to encode input data into a simpler form called latent space (with the capacity to decode them back to reconstruct the original input), typically useful for reducing complexity in data to spot biologically meaningful patterns or to facilitate the manipulation of the latent features (e.g., introducing controlled changes) and noise removal. In contrast, a VAE introduces a probabilistic framework by modeling the latent space as a distribution, typically Gaussian, and learning parameters (mean and variance) for this distribution. This probabilistic nature makes VAEs particularly suited for generative modeling tasks such as the de novo design of inhibitors and proteins. For further in-depth discussion of DL, readers are encouraged to refer to more specialized resources [34,35].

AlphaFold2 (AF2) [36] seems to be the first successful adoption of transformers (Fig. 2E) into structural biology from the fields of natural language processing. The architecture got subsequently implemented in different tasks such as binding site prediction [37], molecular docking [38], and de novo design of molecules [39].

A key advantage of discriminative models is their ability to achieve high performance by automatically learning relevant features from minimally processed data in an end-to-end manner. A prominent example of such approaches is graph neural networks (GNNs) (Fig. 2F), which model molecules or proteins as graphs representing atoms or residues as nodes and bonds or spatial contacts as edges. During training, nodes iteratively exchange information with their neighbors through message-passing mechanisms that include features such as atomic charges, masses, and hybridization states. This process results in a learned representation of the entire graph that captures both local and global structural relationships. The resulting graph-level representation (also called embedding) is subsequently mapped to the target label or value to be predicted. This architecture is widespread in cryptic pocket identification, atomic interaction predictions, and ligand-based discovery.

In contrast to discriminative models, generative DL models, such as diffusion models, learn the underlying data distribution to generate novel samples that resemble, but are not identical to, the training data. These models synthesize new data by capturing statistical patterns within the input distribution. However, some architectures (like generative adversarial networks [Fig. 2H]) blend both types of DL architectures. Their capabilities stem from building a probabilistic understanding of the data, which enables these models to generate outputs that are statistically similar to the training data, while a discriminator model is continually updating the generator to enhance its output. AlphaFold3 (AF3) [40] is one of the state-of-the-art diffusion models (Fig. 2I) in biology where the models are trained to reconstruct biomolecular structures from noise.

### DL is transforming structural biology

DL has revolutionized structural biology since the introduction of AF2 [36]. With the availability of large datasets and improved computational power, DL supplemented biological sciences with a powerful toolbox to analyze biological data and identify new potential targets and assess their druggability (see Target Structure and Druggability). The contributions and applications of DL pipelines have expanded in unprecedented ways, providing crucial guidance for experimental approaches in modeling complex structures [41–45]. Subsequent development of DL tools has led to the proliferation of all-atom frameworks that can model macromolecular assemblies (see the “Modeling macromolecular assemblies” section) such as RoseTTAFold All-Atom [46], AF3 [40], Chai-1 [47], and Boltz-1 [48]. Similarly, artificial intelligence (AI)-guided drug discovery is advancing rapidly, demonstrating high potential and promise through numerous successful outcomes (recently reviewed in [14,15,49]).

Integrating DL pipelines into structural biology can significantly improve drug discovery outcomes and reduce costs [50]. A large-scale academic collaboration project that used the AtomNet AI framework (a convolutional neural network [CNN]-based pipeline) addressed 318 protein targets in hope of identifying small-molecule inhibitors [51]. The approach found hits to almost every protein class without manual “cherry-picking”, demonstrating its viability as an alternative to HTS with a 7.6% overall hit rate. Given the increasing integration of DL into structural biology and drug discovery, we provide an openly accessible, monthly updated, noncomprehensive list of promising open-source DL models in the field, enabling easier tracking of methodological advances (https://www.kwanlabusyd.com/overview). It should be emphasized that the adoption of DL approaches in the field of structural biology and drug discovery is still evolving (Box 1), and the common drawbacks associated with these methodologies have been briefly reviewed in several publications [11–13,15,16,30,52].

Box 1. What current DL models can and cannot do?

ML/DL models are fundamentally statistical models rather than possessing any form of superpower. It is, unfortunately, common within the scientific community to assume that DL models can generalize to all biological sequences. In reality, such models can only extrapolate and accurately predict sequences or structures that share considerable similarity to their training data. Determining the nature and extent of this similarity remains a challenge, especially considering the field’s infancy. A retrospective look for 4 years illustrates this point: we believed that the protein folding problem was solved by AlphaFold2 [36] due to its many accurate predictions, which later matched experimental results [41]. Similarly, impressive results from diffusion models or DL-guided docking models were reconsidered after issues with similarity-based data leakage were highlighted—this problem extends even to small molecules and binding pocket similarities [53,54]. Therefore, if a model predicts the 3D structure of a complex deemed “novel”, this does not necessarily mean that it has “mastered” the chemistry and physics of biological interactions; more likely, the model has identified a form of similarity that we have yet to recognize or detect.

Currently, a handful of well-established DL models have revolutionized how we do science [40,46–48,55]. First, DL models can accurately predict the 3D structures of biomolecules only when they share some similarity with instances in the model’s training data [56]. The accuracy of the prediction is proportional to the similarity level between the query and the training data. In contrast, low-abundance proteins or those from underrepresented families in biological databases are less accurately modeled [57].

Second, present-day co-folding DL models [40,46–48,55,58] and de novo design models [39,59–62] are useful for generating hypotheses about interactions, guiding experimental design or downstream analysis, although these predictions are still imperfect.

Lastly, the most successful DL applications are currently found in ligand-based virtual screening and molecular property prediction, where efficient frameworks and published success stories are frequently published [10,63]. Incorporating physics-based constraints has led to notable improvements—such as chirality conservation in Boltz-1x and physically plausible ligand poses in NeuralPLexer predictions—which are expected to expand into further applications.

## DL Enhances the Target Identification Process

### Key attributes of bacterial drug targets

For a protein to be a suitable target for the development of an antibiotic, it needs to meet some essential criteria. First, it should be essential to bacterial survival or virulence with little or no redundancy in order to inhibit bacterial growth or to block pathogenesis. The essentiality of a gene can vary depending on the growth conditions as a “nonessential” gene in one environment might be essential in another [64,65]. Low redundancy is required so that the bacterium cannot easily compensate by using an alternative pathway or an isoform for the modulated target, thus reducing the chance of rapid resistance development. Second, it should have no close homologs in humans to avoid off-target binding and toxicity issues. The level of similarity required to be considered as “close” to humans’ counterpart protein is generally hard to define. For the most part, at least the key residues at the binding site need to be different. Third, the target must be druggable. The term “druggable” varies by context; a shallow and wide binding site might not be suitable for small-molecule drug discovery but could be appropriate for peptide-based approaches [66]. The presence of cryptic pockets may facilitate the modulation of proteins previously believed to be undruggable.

Numerous virulence factors have been extensively studied and experimentally validated as key contributors to pathogenicity, thereby presenting them as potential drug targets [67]. Despite their therapeutic potential, this area remains relatively underexplored, largely because traditional approaches are mostly focused on compromising bacterial viability. Targeting virulence is expected to impose a reduced selective pressure on bacteria, which may, in turn, lead to lower mutation rates and slower resistance development. While some virulence factors are essential structural components, such as secretion systems that might exist in nonpathogenic species as well, many others are specific and function as regulators of pathogenic pathways, including toxins, quorum-sensing proteins, and biofilm formation modulators [68].

### Target essentiality prediction

Traditionally, gene essentiality was assessed by expensive and laborious laboratory experiments that involved survival quantification or other phenotypic changes after introducing mutations to the gene of interest [69,70]. Consequently, computational approaches to predicting essential genes emerged as more biological data became available. Currently, the common approaches are based on ML or DL models that incorporate different types of genomic and molecular data [71].

DeeplyEssential [72] seems to be the only and carefully implemented DL model to predict bacterial essential genes based on genomic and protein sequences retrieved from the Database of Essential Genes [73]. The model is a typical feedforward neural network trained on features extracted from a balanced dataset of labeled genomic and protein sequences. Although some of these features are highly correlated, the implemented regularization (dropout) seems to have mitigated the risk of overfitting. However, the removal of highly similar genes (~50% of the balanced dataset) decreased model specificity from 0.72 to 0.65, which decreases the model’s accuracy and capability to generalize to new sequences.

Most of the published DL models to predict gene essentiality focus on human genes, but their technical methodologies can also be adopted to predict essential genes in bacterial pathogens. For instance, DeepEP [74], EP-EDL [75], and DeepHE [76] are frameworks that use protein–protein interaction networks or position-specific scoring matrices as inputs to predict protein essentiality. It should be noted that gene or target essentiality prediction should be applied as an initial prioritization tool rather than a conclusive determinant of target suitability for drug discovery.

### Challenges in predicting essential targets

Despite the availability of databases and resources regarding essential genes [77], data quality is the main pitfall, specifically the lack of labeled data derived from similar assessments under identical conditions [78,79]. A few studies have attempted to predict essential protein-coding genes in bacteria using classical ML models trained on balanced datasets containing features derived from sequences and cellular localization [80–82]. However, data leakage is highly suspected as no attempt was made to ensure that homolog proteins or genes in the dataset are not scattered in the training and test data subsets. In contrast, other studies [83–85] used clustering methods on homologous proteins to remove redundant sequences from their datasets, hence avoiding data leakage and its consequences.

Class imbalance and high data dimensionality are also critical challenges for target essentiality prediction models. Ideally, essential genes (positive samples) should not be significantly outnumbered by nonessential ones, but datasets are often highly imbalanced. While different approaches for downsampling the majority class were employed in different models, data loss is almost inevitable [86]. Oversampling of the minority class by methods such as synthetic minority oversampling technique or the adaptive synthetic approach are not recommended options as they introduce risks related to synthetic data generation, including data leakage and distributional assumptions. Managing high-dimensional data is also essential for building efficient DL models. Traditional dimensionality reduction methods such as principal component analysis [87], t-distributed stochastic neighbor embedding [88], and uniform manifold approximation and projection [89] are commonly used. More recently, self-supervised DL models like autoencoders have proven effective in learning compact, informative feature representations [90–92], hence less detrimental data loss during reduction.

## Target Structure and Druggability

### Modeling macromolecular assemblies

The identification and validation of a target’s 3D structure is pivotal for subsequent hit discovery processes. Experimental methods such as x-ray crystallography, nuclear magnetic resonance spectroscopy, and cryogenic electron microscopy have been the primary source for structural models of targets for structure-based drug discovery (SBDD). However, the laborious nature of such methods necessitates the development of computational alternatives based on the accumulated publicly available experimental data. The classical comparative modeling (via homology modeling, fold recognition, or ab initio methods) was the standard computational alternative before the introduction of DL models. The breakthrough of AF2 facilitates the prediction of targets’ 3D structure with an accuracy approaching the experimental level [36].

**Fig. 3.**
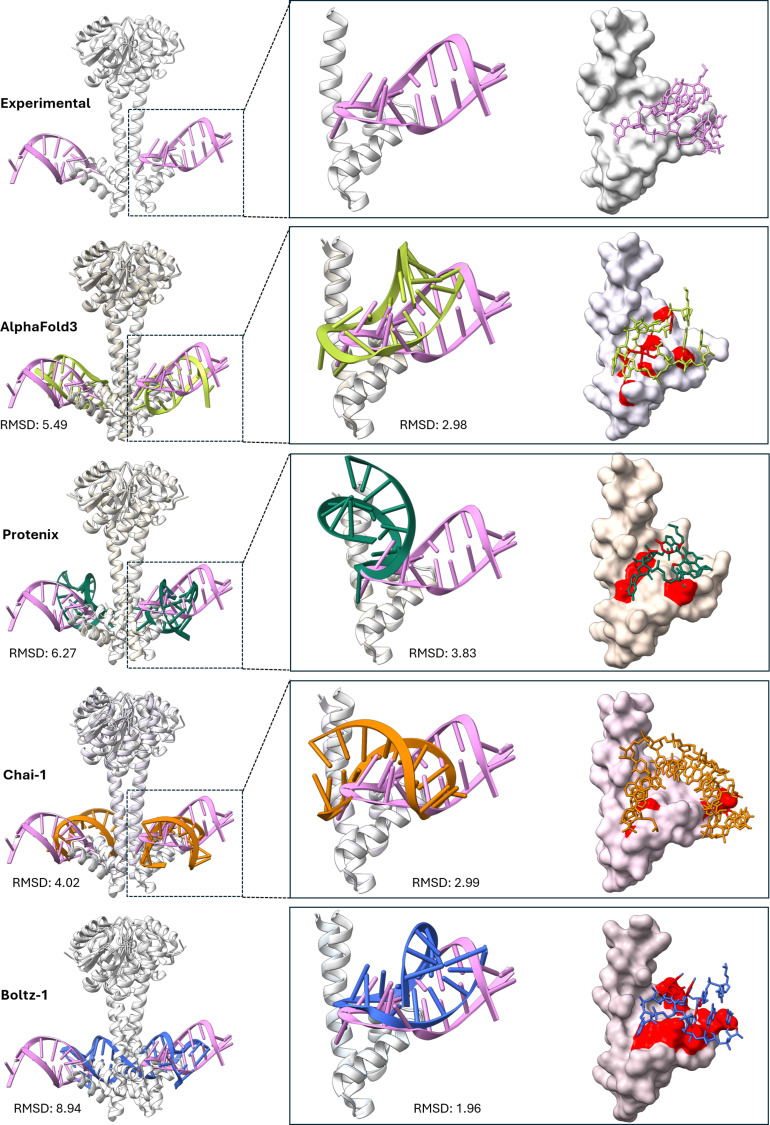
A simple evaluation of different DL models using the ANTAR antiterminator domain (Protein Data Bank [PDB] ID: 6WW6 [https://www.rcsb.org/structure/6WW6]) as a test structure. The experimentally solved structure is shown in the left column as a reference on which the predicted models with their partner RNA chains are superimposed. The middle column is an inset view of the RNA-binding region that shows the overall fold of the RNA and its binding to the protein. The right column shows RNA intramolecular clashes (red sticks) or protein atoms (red patches on the surface). The root mean square deviation (RMSD) values are for superimposition of protein backbones. The lower panel of the Boltz-1 model was created from a prediction based on one monomer as the RNA molecules in the dimer model were predicted as a double-stranded RNA (shown in lower left).

The architecture of AF2 inspired the development of RoseTTAFold All-Atom [46], the first model predicting the 3D structures of nucleic acids, small molecules, and ions in their bound state with protein partners. The recent advent of AF3 [40] and other all-atom 3D structure prediction pipelines, such as Protenix [55], Chai-1 [47], and Boltz-1 [48], has exponentially increased the number of structural hypotheses that can be generated for a given drug target, be it a single protein or a protein–nucleic acid complex. These co-folding approaches theoretically supersede traditional docking methods by accounting for the intrinsic flexibility in the biomolecular complex (see the “Co-folding of the inhibitor with its receptor” section). The confidence scores associated with the predictions made by these pipelines might provide invaluable insights into the overall and local quality of the proposed 3D models. Despite the high accuracy of these models, structural deficits (e.g., atomic clashes and incorrect chirality) can be common in certain assemblies, especially protein–RNA complexes (Fig. 3). Additionally, limited stoichiometry is another issue with space for improvement, especially for heteromeric assemblies.

Prior structural knowledge on the binding site of the target protein might be necessary to select the proper structure for SBDD, especially if a disordered region was modeled to interact with residues of the binding site (e.g., the solved structures of FtsY vs its AF2 model AF-P10121-F1-v4 [https://alphafold.com/entry/P10121]). AF2 structure predictions are guided by constraints learned during training and those in the pair and multiple sequence alignment representations during prediction [36]. Therefore, the predicted structures may lack the optimal architecture of the binding site or even have totally collapsed binding sites to be used for downstream virtual screening studies or pharmacophore modeling. It has been shown that many AF2 structures are more similar to the holo state but proper physics-based refinement of AF2 structures might be required. Such postprediction refinements were found to increase the success in virtual screening [93] or even restores the binding site architecture [94]. On the other hand, AF3 was developed explicitly to predict the holo form where possible [40]. The structures generated by these pipelines have promises for SBDD; however, the binding sites might be modeled inaccurately. The rotamers of the binding site might resemble the architecture of different apo or holo structures used during model training or even during template-based modeling. It would be wise to refine, at least, the rotamers of the binding site to match a high-quality structure co-crystallized with its partner (e.g., ligand, peptide, or nucleic acid) without jeopardizing the binding site quality by introducing atomic clashes or unrealistic orientations.

### Cryptic pockets can be revealed by DL models

Cryptic pockets are transient ligand-binding sites that are not usually visible in static, ground-state structures. It is worth mentioning that there is no universally accepted definition for crypticity. These pockets form due to conformational changes in the protein structure triggered by thermal or pH changes. Targeting these pockets can expand the number of druggable proteins, but the detection methods are still far from being accurate. The determination of cryptic pockets is achieved experimentally or via long molecular dynamics (MD) simulations (or enhanced sampling methods) of the protein of interest with a small-molecule probe (usually benzene, phenol, or imidazole). Although some studies [95,96] have shown that stochastic sampling of AF2 multiple sequence alignment input can lead to predictions that reveal cryptic pockets, it was not successful for all targets [97].

DL-based predictions of cryptic pockets is an area of active research with notable challenges. The datasets of apo–holo conformations for experimentally known proteins that have cryptic pockets are usually small. Consequently, several attempts were made to build larger datasets by using predictive tools to include more examples of cryptic pockets, as seen in the CryptoSite dataset [98]. Unfortunately, these tools also introduce uncertainties (unreal cryptic pockets) owing to their inaccurate predictions. The recently published CryptoBench database [99] loosely define crypticity to include the cavities of ions. However, these sites offer minimal benefit for drug discovery purposes as in the case of metal ions in cytochromes or zinc finger proteins.

PocketMiner [100] is an equivariant GNN model that incorporates equivariance to transformations and predicts the likelihood of residues to form a cryptic pocket. The authors employed adaptive sampling MD simulations to generate the training data. This is particularly useful for learning of physical and molecular systems where geometric constraints must be considered [101]. The model was trained on proteins displaying cryptic pocket opening events, allowing it to identify cryptic pocket residues by modeling residue chemical and topological environments as nodes, with edges representing directions and distances to their 30 nearest neighbors. The promising results are still limited by the small dataset size (*n* = 37) used to train the model, but it represents a sound starting point.

### New opportunities for challenging targets

The diversity of known druggable proteins is increasing due to techniques development and advancing concepts of inhibitor discovery/design, especially when guided by DL techniques [102,103]. Engineered biologics like DARPins and DNA-encoded libraries enable HTS for challenging targets [104]. Likewise, the application of geometric neural networks facilitated the design of inhibitors as seen in the cases of SARS-CoV-2 spike protein and the main protease [105]. Nevertheless, other druggable targets remain challenging, such as RBPs.

RBPs have traditionally been considered challenging drug targets because their RNA-binding regions are often shallow, wide, dynamic, mostly hydrophilic, and context-dependent complexes, all of which pose substantial challenges for inhibitor development. Identifying and targeting specific interactions without off-target effects is difficult because many RBPs can interact with multiple RNA targets via multiple conformations. Nonetheless, inhibitors that compete with RNA to bind human RBPs implicated in various cancers have been reported [106,107]. In contrast, only a few allosteric inhibitors against certain bacterial transfer RNA synthetases have been identified [108].

The current methodologies for studying RNA–protein interactions in vivo are still maturing, with many RBPs being characterized experimentally in the absence of their partner RNA molecules or with missing domains. Furthermore, the inherent structural flexibility of RNA molecules presents challenges for structural studies of such complexes. To address these gaps, various ML methodologies have been developed to predict RNA-binding regions on protein partners. While these models have demonstrated good performance on common RBPs, such as ribosomal RNA- and messenger RNA-binding proteins, their prediction of transfer RNA-binding proteins and novel RBPs remains suboptimal (reviewed in [109]). DL techniques have introduced new avenues for enhancing model accuracy by incorporating the structural details of RBPs. Notable models include NucleicNet [110], GraphBind [111], PST-PRNA [112], GeoBind [113], and NesT-NABind [37]. Despite the superior performance of NesT-NABind (accuracy still <60%), these models continue to face challenges in accurately predicting RNA-binding sites on protein sequences or surfaces, as evidenced by the linear decline in area under the precision–recall curve plots. This issue likely stems from the binary classification approach applied to protein sequences or surfaces, where binding sites constitute a minor fraction compared to nonbinding regions. An efficient approach to overcoming these limitations might involve the adoption of diffusion models, similar to those utilized in blind docking software, which have demonstrated efficacy in identifying novel ligand-binding sites on protein surfaces [114].

## Finding the Potential Hits for a Selected Target

Different DL architectures have been implemented to discover new antibacterial molecules (small molecules and peptides) through ligand-based or receptor-based discovery schemes. The majority of successful examples are ligand-based approaches in which the model is trained to learn the properties of active vs inactive molecules without any input about the target receptor. For receptor-based approaches (molecular docking and co-folding), different benchmarking studies have shown that DL models can outperform physics-based methods in hit identification (distinguishing binders from nonbinders) even with inaccurate prediction of the binding poses [115,116]. On the other hand, target-aware generative DL architecture has shown promising outcomes in different aspects of hit discovery including de novo generation of hits or predicting the binding pose of ligands to their partner receptors.

### Generating ligand 3D conformers is still challenging

Unless the co-folding approach (see the “Co-folding of the inhibitor with its receptor” section) was followed, molecular docking is an important part of the workflow, either for virtual screening or ranking small molecules based on their atomic interactions with the residues at the targeted site. Therefore, generating the correct conformer for a ligand is the first step to start reliable molecular docking and downstream modeling [117]. The conformational space that a molecule can sample is huge and grows exponentially with its size and the number of rotatable bonds. For a molecule with *k* rotatable bonds, sampling its rotation degree of freedom using *N* discrete torsion angles per bond yields *N^k^* conformers. For instance, sampling the 3D conformational space of ampicillin (only 4 rotatable bonds) by an increment of 10 degrees would generate 36^4^ conformers (~1.68 million). Of course, most of these conformations are not in the lowest energy state and therefore unstable, yet the modeling software might need to evaluate these conformers, and the final possible number of low-energy conformers is still high (thousands if only 3 local minima per bond were found). Therefore, stochastic sampling (e.g., Monte Carlo and genetic algorithms) is used to select initial conformations and further optimize and refine them to remove clashes and physical strains. However, as the molecule’s size and complexity increase, these approaches can become computationally intensive or even fail [118].

DL has been applied to the process of ligands’ 3D structure generation at different levels. The early adoption was to use neural networks for geometry optimization of the conformers generated by traditional physics-based methods (experimental-torsion-knowledge distance geometry in RDKit) as seen in the Auto3D pipeline [119]. Subsequent DL models were developed to predict the 3D conformers independent of traditional methods. DMCG [118] and ET-Flow [120] are current state-of-the-art DL models that outperformed traditional methods in 3D conformers’ generation when evaluated on the GEOM-Drugs dataset [121]. However, their performance starts to decline like that of traditional methods when the molecule has >8 rotatable bonds. The methodologies of these models intersect with computational chemistry, which extends beyond the scope of this article.

Although docking programs can alter the 3D structure of the ligands to find the best conformation complementary to the shape of the receptor’s binding site, the complexity of conformational space that the search algorithm needs to explore during docking is huge and the global minimum is difficult to find like the problem of predicting 3D conformers. To evaluate how different initial conformations generated by different methods affect the outcome of docking, conformers of 100 different molecules from experimentally solved protein–ligand complexes in PDBbind were generated by 5 different methods and redocked via Smina (a fork from AutoDock Vina) [118]. The evaluation metrics used were the docking score and root mean square deviation (RMSD) of the top-ranked pose generated by molecular docking against the solved pose. The average docking scores for the conformers generated by the top DL methods DMCG [118] and GeoDiff [122] were superior to those of RDKit.

### Ligand-based approaches

The underlying idea behind a ligand-based approach is that if a molecule has certain substructures and the ability to adopt a 3D conformer that mimics a known inhibitor, then it might be an inhibitor as well for the same target. To efficiently identify small molecules that share similarity with a known inhibitor(s), new types of small-molecule representations have been introduced with DL architectures that outperform classical methods that are based on molecular descriptors and/or connectivity fingerprints [123]. For instance, a message-passing neural network (implemented in Chemprop [63,124]) was trained on HTS results (3,100 molecules) of *Escherichia coli* growth inhibition. When the model was used to screen a larger chemical library (107 million molecules) [10], it led to the discovery of a novel broad-spectrum antibacterial agent called halicin with a minimum inhibitory concentration (MIC) of <10 μg/ml against carbapenem-resistant Enterobacteriaceae and multidrug-resistant *Acinetobacter baumannii* and *Mycobacterium tuberculosis*. A similar approach was also applied to *A. baumannii* [125], and the top hits (abaucin and others) showed growth inhibition for wild-type and resistant phenotypes (MIC: 2 to 10 μg/ml).

To elucidate the mechanisms underlying the prediction of potentially active molecules by DL models, a hypothesis was formulated suggesting that certain chemical substructures largely influence prediction scores. To validate this hypothesis, a Monte Carlo tree search was applied with a selection of a substructure and iteratively trimming part of it and passing the refined subgraphs into Chemprop for scoring purpose. Finally, the deletions that led to smaller substructures with higher prediction scores were identified [126]. Removal of molecules sharing similarity by ≥50% to the training data as well as those containing substructures in known antibiotics led to discovery of a new class of antibiotics. The candidate molecules have activity against gram-positive species (MIC: 2 to 16 μg/ml), and they kill bacterial cells by disrupting the proton motive force across the membrane [10,126].

Predicting AMPs using DL has also become an area of intense research, and success has been seen with different model architectures. The models are developed either to predict the probabilities of AMP activity against target species or to predict the AMP mechanism of action (antibiofilm, membrane disruption, etc.). In iAMPCN [127], one-dimensional CNNs were trained to extract features from different amino acid representations (one-hot, BLOSUM62, AAIndex, and PAAC encodings) to predict AMP and non-AMP probability. Transfer learning was subsequently implemented to predict AMP activity. The incorporation of multiple representations, especially BLOSUM62, facilitates the capture of evolutionary information of AMPs. During cross-validation, the model performance was high (all metrics >0.85) on a dataset containing peptides with ≥50% sequence similarity. However, the performance dropped markedly when the sequence similarity was ≤40% (all metrics <0.63). Two issues can be inferred in this case: (a) systematic data leakage from inadequate separation of homologous sequences and (b) the imbalance problem as reflected by the model’s specificity. Specifically, overlapping sequence motifs or structural features shared between training and evaluation subsets likely enabled the model to exploit similarities rather than learning generalizable discriminative patterns; hence, the model showed excellent performance when the dataset contained ≥50% sequence similarity. Despite employing a focal loss function to mitigate the severe class imbalance (AMP:non-AMP ≈ 1:4), the model exhibited asymmetric performance, with high specificity (0.99) but poor sensitivity (0.51). This asymmetry reflects persistent bias toward the majority class (non-AMP), a well-documented challenge in imbalanced classification [128]. The external test set employed to evaluate the model showed unexpected, good performance. This scenario typically indicates methodological issues with the validation or the final testing process rather than a positive surprise. Population differences between training/validation and external datasets might be implicated as well.

### Receptor-based approaches

DL contributions in SBDD span all stages of the process that starts with receptor structure prediction (discussed in the “Generating ligand 3D conformers is still challenging” section), ligand conformational sampling, virtual screening, scoring functions, and hit optimization. It should be mentioned that all current DL docking models cannot generalize on novel receptors that have no or very low similarity with their training datasets [38]. Despite such ongoing pitfalls, these methods have huge promise in terms of speed, scalability, reducing costs, and accuracy. One of the early successful docking tools that leverage DL capabilities was the Gnina software [33], which continues to evolve over the past 5 years with retraining and implementing new functionalities [129]. Gnina adopted the search algorithm from Vina with the introduction of new scoring functions developed via training a CNN on protein–ligand 3D complexes. This approach retained the physical plausibility of Vina while rescoring the predicted poses. CarsiDock [38], on the other hand, is the first DL-guided docking method to leverage large-scale pre-training on ~9 million protein–ligand docking complexes generated via classical molecular docking. Although this addresses the data scarcity issue that previously limited the accuracy of docking models, biases or inaccuracies in docking results will affect the downstream learning from these complexes. The approach combines distance matrix prediction with geometry optimization to generate binding poses without heavy sampling, a common limitation in traditional docking tools. Despite the fine-tuning of the model on crystalized complexes, physical and chemical violations in the predicted poses were still seen [130]. It is worth mentioning that in terms of enrichment factor, CarsiDock showed the highest performance on the MassiveDecoy dataset that mimics the real-life scenario of virtual screening [116].

To capture intermolecular interactions, CarsiDock predicts atomic distance matrices between proteins and ligands. These predictions are enhanced by a “triangle self-attention” mechanism, an interesting idea adopted from AF2 architecture. The geometry optimization procedure reconstructs the binding pose by refining the translations, rotations, and torsion angles of ligand conformers. In terms of performance, CarsiDock successfully predicted poses with RMSD ≤ 2 Å for 79.7% of the test tasks, and 47.7% of all predictions passed the PoseBusters quality checks (47.7% and 51.2% of Gold and Vina, respectively). It is unclear where and how CarsiDock violates the quality checks set by the PoseBusters suite. However, these observations, coupled with the recent benchmarking [116], indicate that CarsiDock has the potential to significantly outperform classical docking tools and numerous DL docking frameworks. Retraining CarsiDock on a diverse and carefully designed dataset such as PLINDER [53] with considerations of additional quality checks would be promising given its success in generating poses very similar to the solved complexes.

Importantly, pose-centric metrics such as RMSD and physical plausibility checks primarily assess structural fidelity, which is critical when docking predictions are interpreted mechanistically or used for structure-guided optimization. However, these metrics do not necessarily correlate with virtual screening enrichment, ligand ranking, or the practical use of docking as a hypothesis-generation or prioritization tool. In antibacterial discovery workflows, docking is often employed as a triaging step rather than as a definitive predictor of the native bound pose. Consequently, limitations in pose accuracy or physical realism predominantly constrain downstream mechanistic interpretation and lead optimization, rather than initial hit identification.

Diffusion models have received broad interest, especially after the introduction of DiffDock [54], in the prediction of protein–ligand interactions. DiffDock is a generative diffusion model that reframes molecular docking as a generative modeling problem on the non-Euclidean manifold of ligand poses rather than as a regression task as implemented in EquiBind [131] and TANKBind [132] or as a search task in classical docking approaches. DiffDock defines a diffusion (stochastic) process over the 3 classes of degrees of freedom in docking: translation, rotation, and torsion. It learns to reverse the process through the use of an equivariant GNN. This mimics natural binding dynamics, where the ligand gradually “explores” conformations before settling into the optimal pose that favors the receptor’s binding site. The entire process in DiffDock is repeated for multiple steps to naturally explore multiple plausible binding modes rather than collapsing to a single average. After the introduction of PLINDER, retraining and re-evaluation of DiffDock reflected the struggle of predicting correct poses on novel receptors [53].

Notably, receptor local dynamic changes in response to ligand binding are largely ignored by all previously discussed docking tools. DynamicBind [133] is a DL framework that employs a geometric diffusion-based GNN trained to restore distorted conformational states of proteins from AF2 structures. It was also trained to restore morph-like transitions between the apo and the holo states from crystal structures. Using a coarse-grained representation, protein residues were treated as nodes with 2 vectors to store (a) the coordinates and the direction of the residue’s motion and (b) the side-chain torsion angles. The ligand was also encoded as molecular graphs where heavy atoms are the nodes and bonds are edges. Although the model constructs the energy landscape of the protein receptor from its apo state and accordingly adjusts its conformation to suit ligand binding, it should be noted that this is not a large-scale sampling search on the backbone. It is a blind dynamic docking where the initial conformation of the ligand (generated via RDKit) is placed randomly around the receptor, and then the model gradually, and iteratively, rotates and translates the ligand while adjusting its torsion angles to fit the best binding site. Then, the model introduces structural changes in the protein backbone around the ligand via rotation and translation movements of the residues as well as modifying their side-chain angles to optimize the interaction with the ligand. This model represents a great stride in overcoming the limited flexibility of the receptor backbone in classical flexible docking.

### De novo design

DL-guided de novo generation of hits is a promising approach due to not only its ability to shorten the time for hit discovery but also its potential to explore new chemical space beyond the currently known chemical libraries. Nonetheless, this approach has a few drawbacks such as biophysical and/or chemical violations in the generated molecules, low-affinity molecules, lack of in vivo properties of the generated molecules, and synthetic accessibility.

Several regressive and transformer models have been proposed to de novo generate antibacterial hits conditioned on a protein receptor. Examples include DiffSBDD [59], Pocket2Mol [60], ResGen [61], and PocketFlow [62]. DiffSBDD has the advantage of integrating reflection-sensitive GNNs that can distinguish stereochemistry. Although this chemical principle is violated by many generative and docking models, the final evaluation of DiffSBDD did not include it as a metric. DiffSBDD also possesses the capability to redesign ligands from a starting structure, a feature that makes it promising for hit/lead optimization, fragment growing, or linking. Independent benchmarking of de novo design generative models revealed substantial physical violations in the generated poses, especially the interactions involving hydrogen bonds in all tested generative models (including DiffSBDD) [134].

The TamGen pipeline is another ensemble of DL models that conditions simultaneously on receptor and ligand information to generate target-tailored ligand Simplified Molecular Input Line Entry System (SMILES) strings from a scaffold library [52]. TamGen employs a pre-trained transformer model (generative pre-trained transformer-like [GPT-like]), the actual generator of the molecules, receiving an input from the protein encoder module and an assistant VAE model. The latter guides the generation process by providing a sample vector of SMILES embeddings from a predetermined distribution. This architecture successfully designed a handful of inhibitors with low-micromolar MICs against caseinolytic protease P of *M. tuberculosis*. A notable advantage of this architecture is its capacity to allow users to incorporate their own libraries of scaffolds and substructures for the generation of inhibitors tailored to specific targets.

De novo design of AMPs has also been boosted by DL architectures such as recurrent neural network and large language models that showed promising results. Li and colleagues [135] developed AMPd-Up, a multimodel recurrent neural network to generate potentially active short (≤50 amino acids) AMPs. Although generative models do not necessarily require negative data for training since their purpose is to learn and leverage the distribution of the data of interest, overfitting or memorization can still occur and sequence similarity analysis of the generated vs the training instances is one of the metrics used for evaluation. About 95% of the generated AMPs by AMPd-Up have similarities between 54% and 69% to the training instances. Despite the currently used level of similarity to exclude overfitting being ≤80%, this cutoff seems to be high given the overfitting seen in other cases when the protein sequence approached such a cutoff [136]. Like co-folding models, the performance declines significantly once the similarity level between the test data and training datasets is reduced [57]. Moreover, AMPd-Up bias toward “LLKK” and “LKKL” motifs in the generated peptides might reflect the fact that these motifs were common in the training data. It was previously shown that repeating units of these motifs are required to disrupt bacterial membranes and show bioactivity [137].

Conversely, a framework of DL models, SeqGAN and Bidirectional Encoder Representations from Transformers (BERT), were employed to generate and select AMPs against both gram-positive and gram-negative model species [138]. SeqGAN is a generative adversarial network that was trained on a compiled dataset of AMPs with known experimental MIC values. To increase the chance of finding active AMPs, the newly designed sequences are forwarded to a classification (BERT-MLP) model trained to distinguish active from inactive AMPs. The structures of potential peptides were predicted by AF2, and their stability was assessed via MD simulations. Nuclear magnetic resonance validated the in silico results, and the top peptides showed MICs of 8 to 16 μg/ml against *Bacillus subtilis*, *Stenotrophomonas maltophilia*, and *P. aeruginosa*. However, the negative data (inactive AMPs) used to train the models were generated via the AmpGram classifier, whose best precision was ≤0.82.

### Co-folding of the inhibitor with its receptor

Predicting an accurate model for protein–ligand complexes has been the central challenge in molecular docking. Most molecular docking software treats ligands as flexible molecules, whereas the receptor is kept rigid. The introduction of flexible docking—where the side chains of the receptor at the binding site are allowed to move—has increased the success rate of predicting reliable poses for certain targets [139,140], yet a comprehensive evaluation in different protein targets is lacking. Despite ligand-binding events in real-life biology being known to induce structural changes in the target backbone to varying extents, flexible docking is still typically limited to a single conformation as the backbone of the receptor remains fixed. While MD simulation and normal mode analysis can produce ensembles of conformations, these methodologies are constrained by computational costs or their inherent limitations in capturing large-scale conformational changes.

In September 2022, DL introduced an innovative solution to the previously described limitations of molecular docking by co-folding the receptor with its interacting ligand, a strategy that can predict orthosteric and allosteric inhibitors. The first co-folding DL model was NeuralPLexer, a biophysics-constrained diffusion model that predicts an ensemble of protein–ligand complexes [141]. Given a protein sequence and a ligand SMILES, a GNN guided by biophysical constraints encodes the inputs into tensor representations. The atomic-scale constraints (distance distributions and contact maps between the protein and the ligand) are derived from homologous structures (experimental or computational) by a pre-trained protein language model. This framework processes input atoms in a unified representation with an attention mechanism to model the interactions. The input tensors are combined with predicted residue-level constraints, and both are forwarded to a diffusion generative model that iteratively samples plausible conformations while respecting physical constraints.

Recently, all-atom diffusion models such as RoseTTAFold All-Atoms [46], AF3 [40], Chai-1 [47], and Boltz-1 [48] emerged with the ability to co-fold the receptor and its potential modulator. However, the ability of these modeling pipelines to predict structures that are different from their training data can sometimes be overestimated. These models were originally evaluated on different benchmarking datasets that focus on macromolecules’ overall fold and RMSD differences, while the similarity of small molecules and binding sites were not considered. Their accuracy of protein–ligand co-folding has recently been found to be correlated with the number of similar complexes used for training these pipelines [57]. The success rate for predicting a protein–ligand structure dissimilar to the structures seen during the training process was less than 20% for AF3, Protenix, Chai-1, and Boltz-1. Even if the receptor and the binding site share >60% similarity with a structure in the training data, the model could not generalize to a ligand-binding pose not seen previously.

Although AF3 is the state-of-the-art framework for protein–ligand complex folding, additional recent benchmark study raised alarming findings [142]. The evaluation involved challenging tasks designed to test the extent to which AF3 has learned the physics and chemistry of ligand binding. The adenosine triphosphate (ATP)-binding protein FtsE (Protein Data Bank ID: 8X61 [https://www.rcsb.org/structure/8X61]) was one of the test complexes, and it was expected that AF3 had “seen” the ATP molecule in diverse binding sites and “learned” its underlying pattern of interactions with different proteins. When the key residues around the crystalized ATP ligand (<3.5 Å) involved in salt bridges, hydrogen bonds, and π-stacking interactions were mutated to glycine and phenylalanine (to occupy the binding site space) or even substituted by other amino acids with completely opposite physicochemical properties, AF3 still predicts the ATP pose similar to the pose seen in the solved structure (RMSD < 2 Å) of the parent sequence. It seems that AF3 has learned (or somehow memorized) the overall spatial arrangement of molecules in biological assemblies but not the proper chemistry of interactions and their plausibility. The reason why AF3 violated the principles of physical interactions remains unclear. Similarly, protein–peptide modeling seems to be another hurdle for DL frameworks. AF3 modeling of protein–peptide similarly to protein–ligand interactions showed inaccurate modeling (RMSD ≥ 4.5 Å) or even placed a hydrophobic tetrapeptide in a highly positively charged pocket [142].

## Current Challenges Faced by DL Models

### Interpretability and structural difficulties

One major issue for the adoption of DL algorithms in many biosciences is the opacity of these systems. The learning process in neural networks resembles the one in humans by being unclear or extremely difficult to explain. Due to the complex data structure, learning process, and extensive mathematical computations that occur within the hidden layers of deep neural networks, numbers that do not look like your input may shuttle back and forth between layers before the final prediction. As a result, neural networks are often perceived as “black boxes”.

While AF3 and counterparts have revolutionized structure prediction, their outputs are average conformations that may not reflect drug-binding states. Steric clashes, particularly in protein–RNA complexes, highlight unresolved challenges in modeling flexible systems. Additionally, these tools generate confidence scores for every prediction to guide the user in making decisions. However, these scores might be misleading as well [142]. Co-folding of cyclic peptides is still a challenge for these models, and modified residues in such peptides, especially in drug discovery scenarios, add extra complexity.

### Training data suboptimal quality

Data imbalance remains an inevitable issue, especially when the generation of new data is impractical or costly. The use of synthetic data via oversampling to address class imbalance in structural biology and drug discovery is scientifically problematic. While oversampling improves model performance metrics, it does not ensure scientifically valid or biologically meaningful samples, especially in bioactivity prediction tasks. Additionally, bioactivity datasets suffer from inherent experimental biases including differences in assay conditions, measurement (IC_50_, EC_50_, *K*_d_, *K*_i_, or % inhibition), and data aggregation from heterogeneous sources introduce noise that persists despite normalization attempts [143,144]. This limits model generalizability, particularly when predicting binding affinities or bioactivity across diverse protein families (e.g., G protein-coupled receptors [GPCRs] vs RBPs). Similar to classical docking, most DL models for protein–ligand interaction prediction rely on static crystal structures, ignoring dynamic processes such as induced-fit, allostery, and conformational selection. While DL excels at interpolating within known structural space, its ability to extrapolate to unseen conformational states remains limited. Although co-folding tools could have potentially addressed this issue, their learning process is influenced by a combination of apo and holo states. Consequently, predicting the holo form observed upon ligand binding remains a challenge [145].

### Limited generalizability to novel structures

Model performance declines when applied to protein families outside the training set (e.g., enzymes and GPCRs) or novel targets with divergent structural motifs [56,142]. This reflects the scarcity of diverse training data and the vast, underexplored chemical space available. Generative models, although promising, cannot yet navigate this space efficiently due to dataset biases (e.g., overrepresentation of certain chemotypes) and the lack of ground-truth bioactive compounds for rare targets. Furthermore, mislabeled or irrelevant entries (e.g., AMP datasets contaminated with anticancer and spermatocide peptides) exacerbate noise and reduce model reliability [127,136].

## Outlook and Recommendations

DL offers notable advantages over traditional computational methods in structural biology and antibiotic discovery. Its capacity for large-scale macromolecular structure prediction, combined with the ability to screen large chemical libraries, has created opportunities to target new and challenging chemotherapeutic targets. DL-driven de novo design of molecules and peptides further enhances drug development by enabling the creation of novel scaffolds. These new capabilities are expected to significantly reduce the cost of preclinical phase. In specific instances, such as the discoveries of halicin and abaucin, DL has reduced costs to levels not exceeding a few hundred thousand dollars (in contrast to an estimated average of US$31 M per anti-infective drug [146]). However, it remains uncertain whether this trend will be consistent across a broader range of targets. Despite these advances, several challenges remain, particularly as rigorous benchmarking becomes more widespread (see Current Challenges Faced by DL Models). Addressing these issues requires a multidisciplinary approach, engaging both experimental scientists and DL developers.

First, structural biologists play a pivotal role by providing high-quality structural data. This can be achieved by integrating experimental restraints, such as mutagenesis-derived constraints, into MD simulations to generate conformational ensembles to be deposited along with static structures to better capture molecular flexibility.. The public availability of these ensembles, for example, via the Protein Data Bank, derived by a standardized set of protocols [147], would reduce interstudy variability and enable the creation of consistent datasets for training DL models focused on macromolecular dynamics. While initiatives like MDverse and ATLAS represent important progress, challenges remain. For example, MDverse struggles with standardization, search functionality, and metadata reporting, while ATLAS lacks diversity, particularly missing MD simulations of protein–nucleic acid complexes. Additionally, researchers often share data inefficiently, as large amounts of MD simulation data are dispersed across various repositories, frequently as supplementary materials to publications. Creating a centralized, easily accessible, curated, and sustainable data bank for MD simulations would greatly benefit structural biology, drug discovery, and other fields.

Second, variability in bioactivity data across and within laboratories underscores the difficulties in reducing data inconsistencies and noise. The use of standardized assay conditions if possible, or a range of conditions by multiple methodologies, and detailed reporting can increase the confidence in and quality of biological data. These measures would decrease data noise, thereby enhancing model accuracy and generalizability. Comprehensive metadata and reporting standards during dataset depositions would further aid curators in identifying and correcting or mitigating biases.

Third, curators of databases need to prioritize diversity in protein–ligand interactions over sheer dataset size. Overrepresentation of homologous sequences, similar binding sites, or chemical scaffolds can introduce bias and overestimate model performance. Careful dataset curation and preprocessing by curators and DL developers are critical steps to ensure the robustness and reliability of training data.

Fourth, hybrid approaches that integrate DL with physics-based methods can address the unrealistic predictions often seen in purely data-driven models. Incorporating force fields and scoring functions grounded in physical principles such as accurate pairwise potentials and kinetic descriptors implemented in Boltz-1x can improve both interpretability and predictive power [48]. Inaccurate atomic interactions can also be corrected by fusing the training data with physics-informed features.

Finally, sequence-based features, including hidden Markov model matrices supplemented with Gene Ontology-derived data, may serve as additional effective inputs for DL models aimed at predicting essential bacterial genes. Together, these strategies underscore the importance of collaboration across disciplines to fully harness the potential of DL in structural biology and antibiotic discovery.
